# Temperature-Dependent Domain Dynamics and Electrical Properties of Nd-doped Bi_4_Ti_2.99_Mn_0.01_O_12_ Thin Films in Fatigue Process

**DOI:** 10.3390/ma11122418

**Published:** 2018-11-29

**Authors:** Wanli Zhang, Yanhu Mao, Shaoan Yan, Yongguang Xiao, Minghua Tang, Gang Li, Qiangxiang Peng, Zheng Li

**Affiliations:** 1 School of Electronic Information Engineering, Yangtze Normal University, Chongqing 408100, China; zhangwl@yznu.cn (W.Z.); yanhumao@163.com (Y.M.); 2 School of Mechanical Engineering, Xiangtan University, Xiangtan 411105, China; 3 Key Laboratory of Key Film Materials & Application for Equipments (Hunan Province), School of Materials Science and Engineering, Xiangtan University, Xiangtan 411105, China; mhtang@xtu.edu.cn (M.T.); ligangxtu@126.com (G.L.); qxpeng@xtu.edu.cn (Q.P.); lizheng@xtu.edu.cn (Z.L.); 4 Hunan Provincial Key Laboratory of Thin Film Materials and Devices, School of Materials Science and Engineering, Xiangtan University, Xiangtan 411105, China

**Keywords:** domain wall, domain pinning, temperature-dependent fatigue

## Abstract

Bi_4_Ti_2.99_Mn_0.01_O_12_ (BTM) thin films with different ratio of neodymium (Nd) doping were prepared on Pt(111)/Ti/SiO_2_/Si(100) substrates through a sol-gel method. The effects of Nd doping on domain dynamics and temperature-dependent fatigue behaviors of BTM thin films were systematically studied. The polarization fatigues of BTM (not doped) and Bi_3.5_Nd_0.5_Ti_2.99_Mn_0.01_O_12_ (BNTM05) thin films first get better with the increasing temperature (*T*) from 300 to 350 K and then become worse from 350 to 400 K, while Bi_3.15_Nd_0.85_Ti_2.99_Mn_0.01_O_12_ (BNTM85) thin films show enhanced fatigue endurance from 300 to 400 K. It can be shown that the long-range diffusion of oxygen vacancies in BTM thin film happens more easily through the impedance spectra analysis with *T* from 300 to 475 K, which can be verified by a lower activation energies (0.13–0.14 eV) compared to those of BNTM05 and BNTM85 (0.17–0.21 eV). Using a temperature-dependent piezoresponse force microscopy (PFM), we have found more responsive domain fragments in Nd-substituted films. The microscopic domain evolution from 298 to 448 K was done to further explain that the domain wall unpinning effect has been enhanced with increasing *T*. The correlation between microscopic domain dynamics and macroscopic electrical properties clearly demonstrates the effects of charged domain wall in Nd-doped BTM thin films during the fatigue tests.

## 1. Introduction

The Bi_4_Ti_3_O_12_ (BIT)-based layered ferroelectric thin film has always been one of ferroelectric materials with the most potential to replace the commercial (Pb,Zr)TiO_3_ (PZT)-based ferroelectric random access memory (FRAM) for its high curie temperature, large remnant polarization, and good anti-fatigue properties [1,2,3]. Until now, many works on Nd-substituted BIT thin films, such as Bi_3.15_Nd_0.85_Ti_3_O_12_ (BNT) thin films have been carried out [2,3,4]. Large remnant polarization (*P*_r_) and imprinting, and fatigue-free characteristics have been observed in these thin films. Moreover, A- and B-sites co-substituted Bi_3.15_Nd_0.85_Ti_2.99_Mn_0.01_O_12_ (BNTM) thin films were observed to exhibit enhanced dielectric constant and large *P*_r_ [5]. Mn^4+^ can slow down the transformation from Ti^4+^ to Ti^3+^ and reduces a number of pinned domain walls [6,7]. However, high leakage current and poor fatigue endurance can restrict the application of BNTM thin films with Pt electrodes due to the evaporation of bismuth under high annealing temperature [8,9]. For the purpose of understanding the nature of the fatigue behaviors of BIT thin films, it is necessary to understand how the Nd doping affects the domain dynamics.

Ferroelectric-based memories may operate in temperature range from −40 to 125 °C, which can be essential to understanding the temperature-dependent change of fatigue behaviors of ferroelectric materials. It has been reported that the fatigue endurance for BNT thin films showed improved fatigue resistance from 25 to 125 °C [10], which can be attributed to the fact that domain wall unpinning effect enhances more rapidly with increasing temperatures than that of domain wall pinning effect. Also, the enhanced effect of electron injection can induce pinned domain walls and serious fatigue at high temperatures due to a lower Schottky barrier compared to that at low temperatures [11]. Fatigue behaviors of BNT thin films were also influenced by crystal orientation, with various change trends of temperature-dependent fatigue behaviors for various oriented grains [12]. However, earlier reports mainly studied macroscopic performance tests and neglected microscopic domain dynamics, which are considered to mainly affect the polarization switching and fatigue behaviors. It has been reported that the microscopic domain evolution and activation energies of oxygen vacancies were effectively used to analyze the mechanisms of polarization fatigue in BiFeO_3_ thin films with combinations of impedance spectra techniques, PFM and first-principles theory [13]. Thus, the studies of microscopic domain dynamic and transport law of oxygen vacancies will be helpful to further understand the mechanisms of fatigue mechanisms of BNTM thin films in extreme environments.

In the following sections, polarization fatigue behaviors of Bi_4_Ti_2.99_Mn_0.01_O_12_ (BTM) thin films with different ratio of neodymium (Nd) doping were studied from 300 to 475 K. A combination of temperature-dependent impedance spectra and PFM tests was made to learn the transport mechanisms of oxygen vacancies and microscopic evolution of domain walls in BIT-based thin films. The correlations between macroscopic performance and microscopic domain dynamics of BTM thin films with varying Nd doping at elevated temperature (*T*) were also discussed.

## 2. Experimental

The starting precursor materials were Bi(NO_3_)_3_·5H_2_O, Nd(NO_3_)_3_·6H_2_O, Ti(OC_4_H_9_)_4_ and Mn(CH_3_COO)_2_·4H_2_O. The solvents were 2-methoxyethanol and glacial acetic acid with acetyl acetone as a chelating agent. The concentration of Bi_4_Ti_2.99_Mn_0.01_O_12_ (BTM), Bi_3.5_Nd_0.5_Ti_2.99_Mn_0.01_O_12_ (BNTM05) and Bi_3.15_Nd_0.85_Ti_2.99_Mn_0.01_O_12_ (BNTM85) precursor solutions were adjusted to 0.1 M, and 10% excess of bismuth were added to compensate for possible loss of bismuth during thermal process. These detailed works can be found in our previous studies [14,15,16]. The spin-on films were started at a drying process at 180 °C for 5 min, then conducted at an apyrolysis process at 400 °C for 5 min and an annealing process at 650 °C for 3 min in O_2_. After six repeats, an extra thermal process was given at 720 °C for 5 min in O_2_ for the final layer. Pt top electrodes were deposited with a diameter of 200 µm through DC (direct current) sputtering.

X-ray diffraction (XRD) with Cu Kα radiation was used to study texturing state and crystallographic structure of such thin films. Scanning electron microscope (SEM, Hitachi S4800, Hitachi, Tokyo, Japan) was conducted to characterize the surface and cross-sectional morphologies of these films. Semiconductor device analyzer (B1500A, Agilent, Santa Clara, CA, USA) which was combined with a temperature-controlled probe system was used to measure temperature-dependent dielectric properties and AC (alternating current) impedance spectra of such films. An available commercially Z-View software (version 2.0) was used to analyze the impedance results. Ferroelectric test systems (Radiant Technologies Precisions Workstations, Albuquerque, NM, USA) were used to measure the ferroelectric properties. PFM (piezoresponse force microscopy) tests were conducted by using a combination of AFM (atomic force microscopy) system (MFP-3D, Asylum Research, Santa Clara, CA, USA) and PolyHeater under ambient condition. A platinum coated silicon cantilever (radius: 15 nm, spring constant: 2 N/m) was used to scan with a tip lift height of 30 nm at 35 kHz.

## 3. Results and Discussion

**Figure 1:**
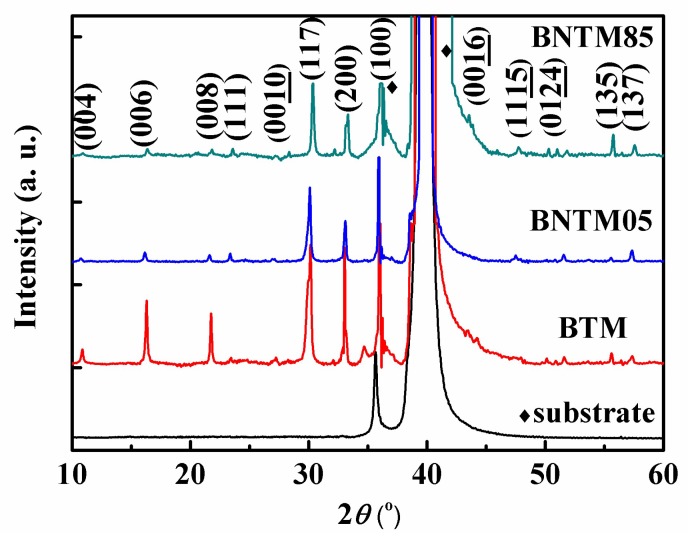
XRD patterns of substrate, BTM, BNTM05 and BNTM85 thin films.

**Figure 2:**
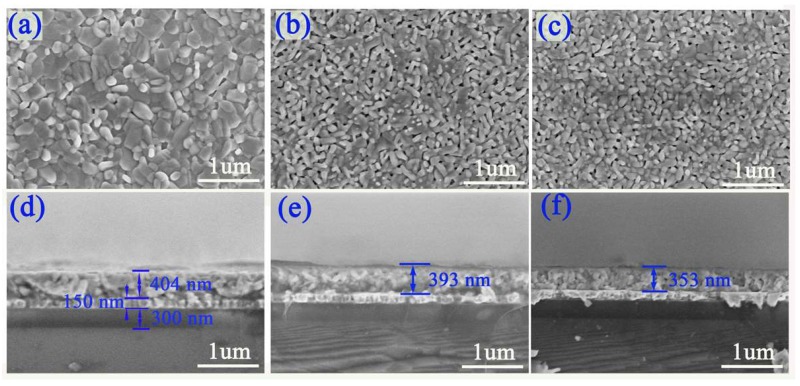
SEM surface and cross-section images: (**a**,**d**) for BTM; (**b**,**e**) for BNTM05; (**c**,**f**) for BNTM85.

A random oriented growth of BTM was observed by XRD as shown in Figure 1, while a (117)-preferred growth was presented in BNTM05 and BNTM85. It has been reported that the structural distortion increases with the decreasing size of the A-site cation and the orientations are strongly affected by structural distortion. The lattice spaces calculated from the (117)-peaks of BTM, BNTM05, and BNTM85 were 2.9727, 2.9684, and 2.942 Å, respectively. Therefore, the structural distortion of doped BTM films was stronger than that of un-doped BTM films, which results in (117)-oriented growth in the BNTM05 and BNTM85 films. The surface and cross-section of such thin films are observed through SEM methods as shown in Figure 2a–g. It can be seen that the BTM thin film shows the largest grains than these thin films with Nd doping. The equiaxed and plate-like grains can be found at the surface of BTM thin films, while the rod-like grains with different lengths are mainly shown in (117)-oriented BNTM05 and BNTM85 thin films (as shown in Figure 2a–c). Film thicknesses of BTM, BNTM05 and BNTM85 were estimated to be 404 nm, 393 nm, and 353 nm through the cross-sectional images shown in Figure 2d–g.

**Figure 3:**
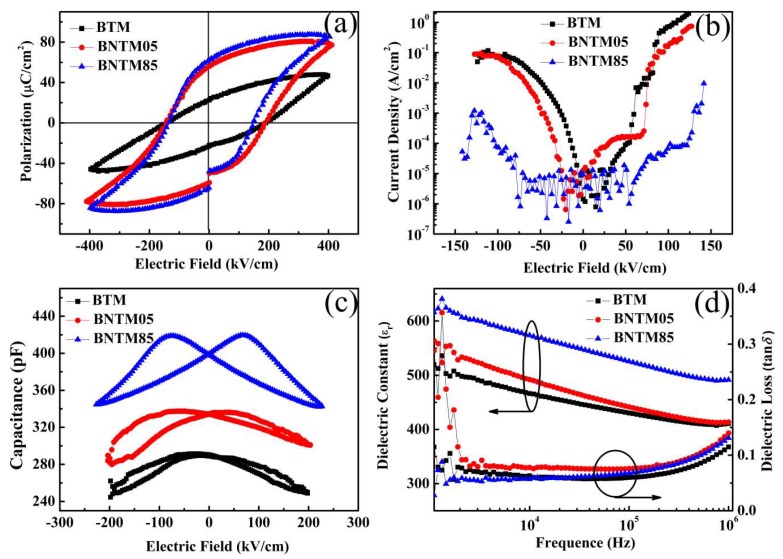
The electrical properties of BTM, BNTM05 and BNTM85 for (**a**) *P*–*E* hysteresis loops at 1 kHz; (**b**) *J*–*E* curves at 6 V; (**c**) *C*–*V* curves at 1 MHz; (**d**) Dielectric constant and dielectric loss curves.

The polarization-electric field (*P*–*E*) hysteresis loops of the three types of thin films were shown in Figure 3a. The remnant polarization 2*P*_r_ and coercive field 2*E*_c_ of such films were found to be about 47.8, 113.8, 128.7 µC/cm^2^ and 163.3, 167.4, 138.8 kV/cm at an applied electric field of 400 kV/cm, for BTM, BNTM05 and BNTM85, respectively. It can be seen that BNTM85 has the highest remnant polarization, the lowest coercive field and the lowest current density compared to those of BTM and BNTM05 as shown in Figure 3a,b of the *J*–*E* curves. The capacitance vs. voltage (*C*–*V*) curves for different types of BTM thin films were plotted in Figure 3c at a frequency of 1 MHz. The highest switching peak (420.0 pF) and dielectric tunability (18.4%) can be found in BNTM85 film compared to the other two types of thin films. The dielectric loss tangent (tan δ) and variation of dielectric constant (ε_r_) were plotted as a function of the measurement frequency for BTM, BNTM05 and BNTM85 in Figure 3d. The dielectric constants and dissipation factors of such films at 10 kHz were 428.2, 443.2, 528.1 and 0.06, 0.08, 0.05, respectively. The largest dielectric constant and the lowest dissipation factor were exhibited in BNTM85 thin films as compared to the others. It is generally accepted that proper Nd doping can prevent the evaporation of bismuth, resulting in an increase of ratio of (117)-oriented polarization, which can provide better electrical properties [17,18].

**Figure 4:**
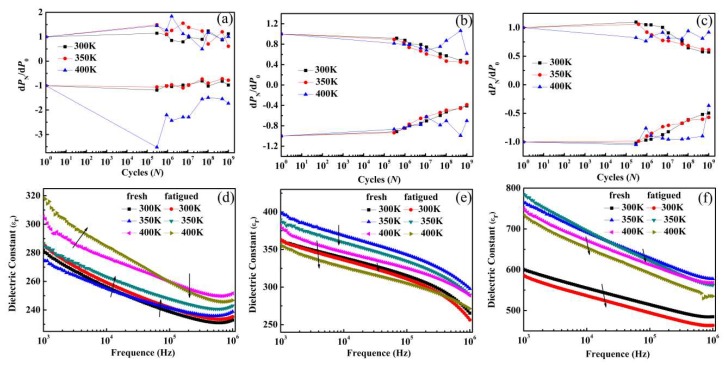
Plots of polarization fatigue curves, ferroelectric hysteresis, and dielectric constant (ε_r_) vs. frequency at both fresh and fatigued condition: (**a**,**d**) for BTM; (**b**,**e**) for BNTM05; (**c**,**f**) for BNTM85.

The fatigue characteristics of BTM, BNTM05 and BNTM85 from 300 to 400 K were displayed in Figure 4a–c. The pulse amplitudes were 10 V and 8 V for reading and fatigue process, respectively. The relationship of ±d*P_N_* = (±*P*_r_^*^)*_N_* – (±*P*_r_^^^)*_N_* can be described that *N* is the number of switching cycles, *P_N_* is the total polarization, *P*_r_^*^ is the switched remnant polarization between the two opposite polarity pulses, and *P*_r_^^^ is the non-switched remnant polarization between the same two polarity pulses. After 1 × 10^9^ cycles of pulse switching, the reductions of d*P_N_ of* BTM, BNTM05 and BNTM85 thin films were 0%, 56.1% and 46.2% at 300 K, 32.0%, 58.4% and 35.9% at 350 K, and 0%, 34.3% and 34.5% at 400 K, respectively. The fatigue characteristics of BTM and BNTM05 thin films become more serious from 300 to 350 K, and improve from 350 to 400 K, while an improved fatigue resistance can be found in BNTM85 thin film with increasing *T* from 300 to 400 K. The improved fatigue properties of BNTM85 thin film from 300 to 400 K should be induced by the enhanced effect of domain wall unpinning [8,14,19]. As for BTM and BNTM05 thin films, the effects of pinning and unpinning of domain walls have been always enhanced from 300 to 400 K. However, the primary factor is the enhanced domain wall pinning effect first and then a more serious fatigue behavior can be induced from 300 to 350 K, while domain wall unpinning effect is the determined role and then an improved fatigue effect from 350 to 400 K can be achieved. It can be concluded that the competition between domain wall pinning and domain wall unpinning has always been an obvious effect on polarization fatigue of BIT-based thin films [20,21,22]. It should be mentioned that the fatigue curves under 400 K clearly fluctuate. Several sources may lead to this phenomenon, such as large perturbation at the tip, enhanced domain unpinning effect and aggregation of long-range diffusion of oxygen vacancies at the film/electrode interfaces.

The plots of dielectric constant (ε_r_) and frequency were further conducted to investigate the dead layer growing effect before and after fatigue process as shown in Figure 4d–f. The value of ε_r_ of BTM thin film increases with *T*, while the values of ε_r_ of BNTM05 and BNTM85 thin films increase with *T* from 300 to 350 K first, and then decrease with *T* from 350 to 400 K. As for BTM, the long-range diffusion of oxygen vacancies becomes enhanced with increasing *T*. After repetitive switching, the aggregation of oxygen vacancies on domain walls contributes to dielectric response and leads to an increase of dielectric constant of BTM. This phenomenon has also been reported in BiFeO_3_ and Pb_0.4_Sr_0.6_TiO_3_ thin films [7,23]. For BNTM05 and BNTM85, the thickness of the dead layer increases with *T,* leading to the reduction of ε_r_ from 350 to 400 K. This change was also discussed in some other works [21,22,23]. Interestingly, the value of ε_r_ of BTM thin film increases when the frequency is changed from 1 to 100 kHz after 10^9^ pulse switching cycles. Similar results were also reported by Ke et al. [24,25]. It was explained that a large amount of charged domain walls formed by oxygen vacancies’ migration at interfaces during the fatigue process had taken part in the dielectric response, which caused the increase of ε_r_ for BTM.

**Figure 5:**
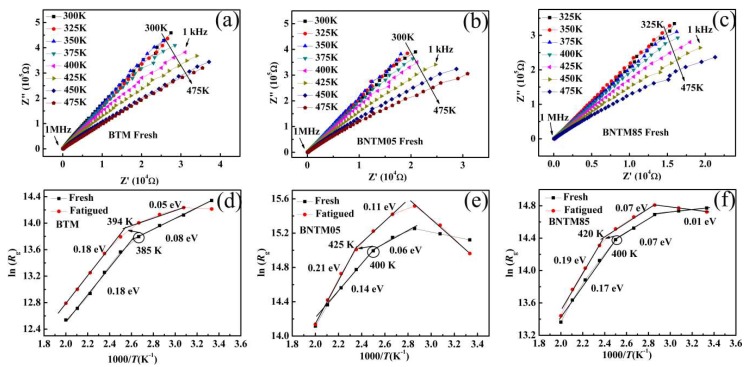
Impedance diagrams at elevated temperature and ln(*R*_g_) vs. 1000/*T* Arrhenius plots at both fresh and fatigued condition: (**a**,**d**) for BTM; (**b**,**e**) for BNTM05; (**c**,**f**) for BNTM85.

AC impedance spectra tests were used to study the conductance mechanism before and after fatigue process with the temperature range from 300 to 475 K. Figure 5a–c shows the real and imaginary impedance (*Z*´ and *Z*”) as frequency decreases from 1 MHz to 1 kHz. The contribution of grain for the conductance can be reflected by high frequency arcs, which was also reported by Pintilie et al. [26]. The nonlinear least square fitting was conducted to estimate resistances of grains (*R*_g_) of BNTM films [27]. The values of *R*_g_ always exhibit a reduction with increasing *T* and become larger after the fatigued process as shown in Figure 5d–f. It can be elucidated that the population of carriers increased with increasing *T* and a part of oxygen vacancies or injected electrons was trapped by charged domain walls during the fatigue process [28]. The *R*_g_ follows Arrhenius’ relationship as $R_{g}\propto \exp\left(-E_{a}/k_{B}T\right)$, where *E*_a_ represents average activation energy of carriers during conductance process and *k*_B_ means Boltzmann’s constant [29]. The curves of ln(*R*_g_) vs. 1000/*T* were shown in Figure 5d–f. The activation energies *E*_a_ can be linearly fitted into two parts for BTM and three parts for BNTM05 and BNTM85 thin films similar to the results presented in our previous work [14]. Negative slope Arrhenius plots can be found in Figure 5e from 300 to 350 K. A lot of interfacial space charges at contacts and grain boundaries of BNTM05 thin films can be removed at low frequencies. The redistribution of these removable charges or defects should escape from the domain walls at the surface of thin films from 300 to 350 K. Thus, the resistances of grains (*R*_g_) of BNTM05 thin films can increase and then negative slopes will occur without the contribution of such removable space charges or defects. Observed activation energies of 0.14–0.21 eV are generally considered as the contribution of long-range diffusion of oxygen vacancies within their clusters in the body of these thin films [24,28], while those of 0.01–0.03 eV can be triggered by partial hopping electrons at the film/electrode interfaces [30], and the values between 0.03 and 0.14 eV are just seen as a combined effect of oxygen vacancies and hopping electrons. It has been found that values of *E*_a_ increase after 1.6 × 10^9^ pulse cycles in all three types of films, which is a certain degree to reflect that more removable carriers should aggregate at the film/electrode interface [28] or at vertical domain walls [31]. Interestingly, the inflection point has transferred from 385 to 394 K for BTM, from 400 to 425 K for BNTM05, and from 400 to 420 K for BNTM85 thin films, respectively. It generally accepted that the inflection is caused by the transition of conduction mechanisms from a combination of hopping electrons and oxygen vacancies to a main contribution from the oxygen vacancies. After fatigue tests, it can be assumed that a part of oxygen vacancies has been redistributed or trapped at the interfaces, which has led to a higher inflection. To some degree, it reveals that long-range diffusion happens more easily for oxygen vacancies in BTM thin film, which leads to a smaller change of the inflection point compared to those in BNTM05 and BNTM85 thin films after fatigue tests.

**Figure 6:**
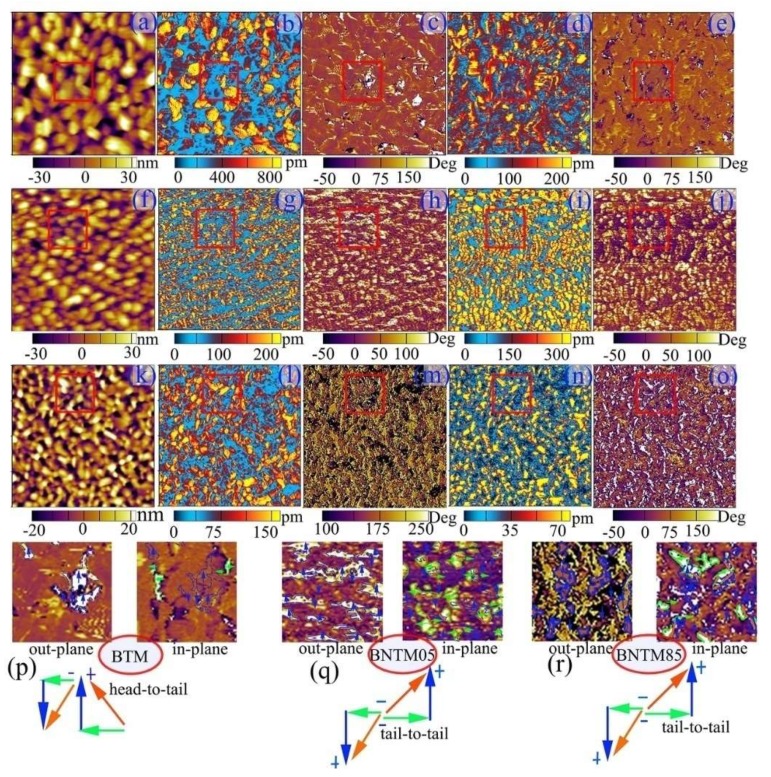
AFM surface topography, OP PFM amplitude images, OP PFM phase images, IP PFM amplitude images, IP PFM phase images and zoomed-in PFM images of specific region in red solid square: (**a**–**e**,**p**) for BTM, (**f**–**j**,**q**) for BNTM05, (**k**–**o**,**r**) for BNTM85, respectively, and the scanning area is 2 × 2 μm^2^.

**Figure 7:**
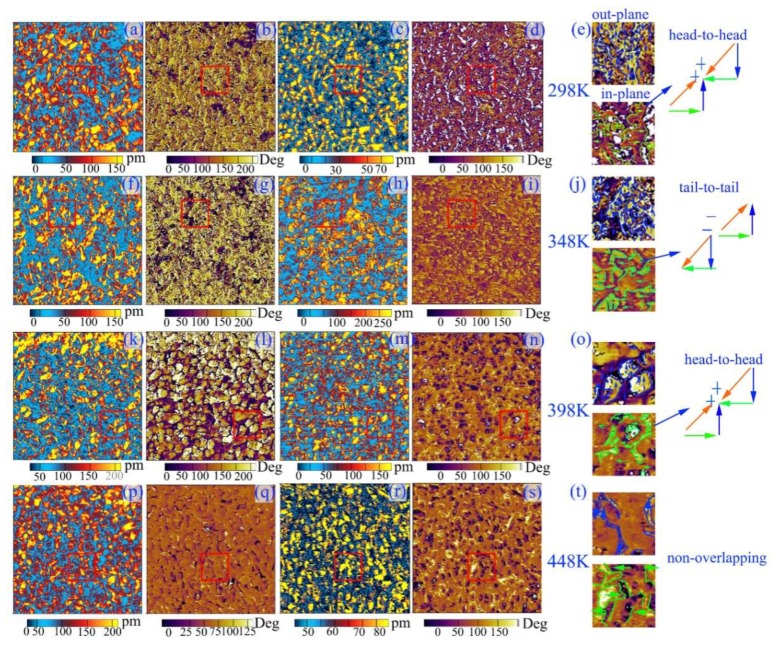
OP PFM amplitude image, OP PFM phase image, IP PFM amplitude images, IP PFM phase images and zoomed-in PFM images of specific region in red solid square of BNTM85 at elevated *T*: (**a**–**e**) at 298 K; (**f**–**j**) at 348 K; (**k**–**o**) at 398 K; (**p**–**t**) at 448 K, respectively. The scanning area is 2 × 2 μm^2^.

The microscopic domain structures of BTM, BNTM05 and BNTM85 thin films were studied through the PFM method. AFM surface topography, OP PFM amplitude images, OP PFM phase images, IP PFM amplitude images, IP PFM phase images and zoomed-in PFM images of specific region in red solid squares of such films were shown in Figure 6a–r. The red solid lines in images of zoomed-in specific region are correlated with the charged domain walls which were caused by head-to-head or tail-to-tail polarization configurations as shown in Figure 6p–r and Figure 7e,j,o,t. When removable carriers such as electrons or oxygen vacancies are trapped by these pinned domain walls, a stable electro-neutral structure will form. The regions with bright yellow and dark colors in OP phase images correspond to vertically up or down 180°-domains, while the regions with rich yellow and dark colors in IP image correspond to laterally left or right 90°-domains. The domain boundaries are marked with blue solid lines for OP images of zoomed-in specific regions, while green solid lines represent the domain boundaries for IP images of zoomed-in specific regions. The overlaying boundaries of the blue lines and green lines are marked with orange red solid lines, which represent the head-to-head or tail-to-tail non-neutral domain walls. It can be seen that the size of domains in BTM thin films are much larger than those in BNTM05 and BNTM85 thin films as shown in Figure 6c,h,m, which was further consumed that Nd doping can bring fragmented domains. It has been reported that fragmented domains break the long-range polar order, and pin the domain wall as found in situ TEM observations [32]. It can be also assumed that a lot of fragmented (117)-oriented domains can facilitate tail-to-tail or head-to-head polarization configurations as shown in Figure 6p,r, which makes the density and thickness of domain wall being a determined role in temperature-dependent fatigue behaviors of BNTM05 and BNTM85 thin films. Thus, the reason why BNTM05 and BNTM85 films show serious fatigue properties can be induced by the higher density of domain walls as compared to those of BTM thin film. In order to study temperature-dependent domains and domain walls in detail, the temperature-dependent PFM observation of BNTM85 thin film was conducted from 298 to 448 K as shown in Figure 7a–t. The amount of charged domain walls which are marked with the red solid lines in the IP PFM images of Figure 7e,j,o,t were decreased with *T* from 298 to 448 K, which indicates that the switchable domains are increasing with *T*. These observations correspond well to the fact that the domain unpinning effect has been enhanced with increasing *T*. It is interesting that the switching domains can merge into a larger one from 300 to 348 K as shown in Figure 7e,j. However, at 398 and 448 K, the higher domain wall velocity can lead to a lower probability of capturing oxygen vacancies, as compared to those at 300 and 348 K for non-neutral domain walls, are shown in Figure 7o,t. Thus, the improved fatigue endurance which happens in BNTM85 at high temperatures can be explained.

## 4. Conclusions

In summary, the influences of Nd doping and temperature on domain dynamics and fatigue behaviors of BTM thin films at elevated temperatures were systematically investigated. The competition between domain unpinning and growth of dead layer induced by the aggregation of removable carriers leads to the different fatigue properties between BTM and BNTM85 at elevated temperatures. It can be shown that the long-range diffusion happens more easily for oxygen vacancies in BTM thin film, while pinned and unpinned domains have determined the fatigue behaviors in BNTM85 thin films. The values of 0.14–0.21 eV of the activation energies should be contributed to the oxygen vacancies with long-range diffusion from 385 to 475 K. The temperature-dependent domain walls with head-to-head or tail-to-tail polarization configurations were observed in the Nd-doped BTM thin films through PFM tests. The interplay between the macroscopic fatigue and microscopic evolution of domain dynamics truly reveals the effects of charged domain wall in the fatigue failure of Nd-doped BTM thin films. These efforts can contribute to our understanding of the temperature-dependent domain dynamics of layered perovskite structure ferroelectric films.
